# TGF-β Induced CTGF Expression in Human Lung Epithelial Cells through ERK, ADAM17, RSK1, and C/EBPβ Pathways

**DOI:** 10.3390/ijms21239084

**Published:** 2020-11-29

**Authors:** Shu-Ching Ou, Kuan-Jen Bai, Wun-Hao Cheng, Jing-Yun Chen, Chien-Huang Lin, Heng-Ching Wen, Bing-Chang Chen

**Affiliations:** 1 School of Respiratory Therapy, College of Medicine, Taipei Medical University, Taipei 110, Taiwan; m141106001@tmu.edu.tw (S.-C.O.); bkj@tmu.edu.tw (K.-J.B.); d119099014@tmu.edu.tw (H.-C.W.); 2 Division of Pulmonary Medicine, Department of Internal Medicine, Wan Fang Hospital, Taipei Medical University, Taipei 110, Taiwan; 3 Graduate Institute of Medical Sciences, College of Medicine, Taipei Medical University, Taipei 110, Taiwan; d119106011@tmu.edu.tw (W.-H.C.); d119104008@tmu.edu.tw (J.-Y.C.); chlin@tmu.edu.tw (C.-H.L.); 4 Respiratory Therapy, Division of Pulmonary Medicine, Department of Internal Medicine, Wan Fang Hospital, Taipei Medical University, Taipei 110, Taiwan; 5 Department of Internal Medicine, School of Medicine, College of Medicine, Taipei Medical University, Taipei 110, Taiwan

**Keywords:** pulmonary fibrosis, transforming growth factor-β, a disintegrin and metalloproteinase 17, connective tissue growth factor, epithelial–mesenchymal transition

## Abstract

Background: Lung epithelial cells play critical roles in idiopathic pulmonary fibrosis. Methods: In the present study, we investigated whether transforming growth factor-β (TGF-β)-induced expression of connective tissue growth factor (CTGF) was regulated by the extracellular signal-regulated kinase (ERK)/a disintegrin and metalloproteinase 17 (ADAM17)/ribosomal S6 kinases 1 (RSK1)/CCAAT/enhancer-binding protein β (C/EBPβ) signaling pathway in human lung epithelial cells (A549). Results: Our results revealed that TGF-β-induced CTGF expression was weakened by ADAM17 small interfering RNA (ADAM17 siRNA), TNF-α processing inhibitor-0 (TAPI-0, an ADAM17 inhibitor), U0126 (an ERK inhibitor), RSK1 siRNA, and C/EBPβ siRNA. TGF-β-induced ERK phosphorylation as well as ADAM17 phosphorylation was attenuated by U0126. The TGF-β-induced increase in RSK1 phosphorylation was inhibited by TAPI-0 and U0126. TGF-β-induced C/EBPβ phosphorylation was weakened by U0126, ADAM17 siRNA, and RSK1 siRNA. In addition, TGF-β increased the recruitment of C/EBPβ to the CTGF promoter. Furthermore, TGF-β enhanced fibronectin (FN), an epithelial–mesenchymal transition (EMT) marker, and CTGF mRNA levels and reduced E-cadherin mRNA levels. Moreover, TGF-β-stimulated FN protein expression was reduced by ADAM17 siRNA and CTGF siRNA. Conclusion: The results suggested that TGF-β induces CTGF expression through the ERK/ADAM17/RSK1/C/EBPβ signaling pathway. Moreover, ADAM17 and CTGF participate in TGF-β-induced FN expression in human lung epithelial cells.

## 1. Introduction

Idiopathic pulmonary fibrosis (IPF) is a type of an interstitial lung disease that is prevalent in elder smokers. The phases of IPF include alveolar epithelial cell damage and activation, inflammatory cell infiltration, epithelial–mesenchymal transition (EMT) initiation, and ECM protein accumulation [1]. EMT is the process by which cells lose their epithelial features and obtain a mesenchymal characteristic. Many growth factors participate in EMT, especially transforming growth factor-β (TGF-β) [2,3]. During the progression of pulmonary fibrosis, most fibroblasts originate from lung epithelial cells, which undergo EMT and play a crucial role in fibrotic disease progression. The TGF-β signaling pathway has been suggested to contribute to the EMT process and produce ECM proteins such as fibronectin (FN) [4]. Therefore, TGF-β and EMT may be a hallmark of fibroblast activation.

A disintegrin and metalloproteinase 17 (ADAM17) is a transmembrane protein that plays a major role in the cleavage of extracellular domains of substrate proteins [5]. In physiological and pathophysiological processes, ADAM17 regulates some important membrane-bound proteins such as cytokines and growth factors [6]. A previous study demonstrated that ADAM17 regulates TGF-β-mediated EMT through cleavage of vasorin [7]. In addition, ADAM17-induced angiotensin-converting enzyme 2 (ACE-2) ectodomain shedding occurred in lung fibrogenesis, demonstrating that ADAM17 certainly participated in pulmonary fibrosis [8]. However, the role of ADAM17 in TGF-β-induced EMT in pulmonary fibrosis remain unidentified.

Connective tissue growth factor (CTGF) is an immediate early protein mediated by TGF-β, and it regulates the growth of fibroblasts and the secretion of ECM [9,10,11]. A previous study suggested that TGF-β subcutaneous co-injection with CTGF induced sustained fibrosis in mice [12]. CTGF protein and mRNA are both enhanced in lung tissue obtained from IPF patients [13]. Moreover, integrin-linked kinase mediates the formation of CTGF-induced EMT in lung epithelial cells [14]. However, the relationship between ADAM17 and CTGF in TGF-β-induced EMT process in human lung epithelial cells remains unclear.

CCAAT enhancer-binding protein β (C/EBPβ), a transcription factor that participates in the modulation of inflammatory protein expression in numerous cell types, is phosphorylated by RSK1, and protein kinase C (PKC) [15,16]. A previous study suggested that the CTGF promoter sequence includes AP-1 and C/EBPβ in zebrafish [17]. In addition, Weng et al. found that MEK inhibitor significantly attenuated CTGF expression in TGF-β-induced renal fibrosis in mice [18], but information on the responsibilities of RSK1 and C/EBPβ in TGF-β-induced CTGF expression in human lung epithelial cells remains limited. In the present study, data showed that TGF-β might activate ERK, ADAM17, and RSK1 signaling pathways, which initiate C/EBPβ phosphorylation and the binding of C/EBPβ to the CTGF promoter region and lead to CTGF expression. Moreover, CTGF participates in TGF-induced FN expression in human lung epithelial cells.

## 2. Results

### 2.1. TGF-β Induced CTGF Expression in Human Lung Epithelial Cells

**Figure 1:**
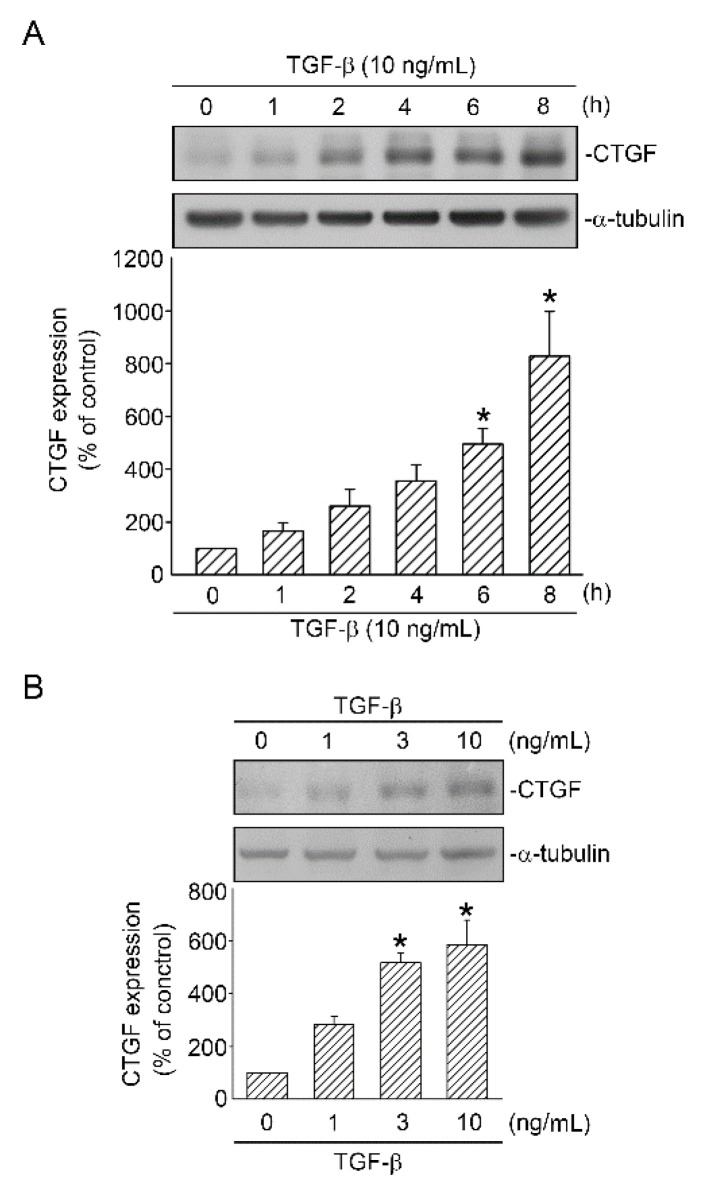
TGF-β-induced CTGF expression in human lung epithelial A549 cells. (**A**) A549 cells were treated with TGF-β (10 ng/mL) for 0–8 h. Cell lysates were prepared, and CTGF and α-tubulin antibodies were detected through Western blotting. Data are expressed as mean ± SEM of three independent experiments. * *p* < 0.05, compared with the control group without TGF-β stimulation. (**B**) Cells were stimulated with TGF-β (0–10 ng/mL) for 6 h. Cell lysates were prepared, and CTGF and α-tubulin antibodies were detected through Western blotting. Data are presented as mean ± SEM of three independent experiments. * *p* < 0.05, compared with the control group without TGF-β treatment.

TGF-β contributes to initiating, maintaining, and amplifying the effects of other cytokines and chemoattractants related to the pathogenesis of lung injury [19]. CTGF is identified as an early phased gene, the expression of which is induced by TGF-β; moreover, its overproduction is suggested to play a crucial role in pathways leading to organ fibrosis [10,20]. In the present study, we investigated whether TGF-β increases CTGF expression in human lung epithelial cells (A549). When A549 cells were treated with TGF-β (10 ng/mL) for 0–8 h, CTGF expression significantly increased at 6 h, with a maximal effect at 8 h (Figure 1A). Additionally, treating A549 cells with TGF-β(0–10 ng/mL) for 6 h in a dose-dependent manner induced CTGF expression (Figure 1B). The results indicated that TGF-β enhanced CTGF protein expression in human lung epithelial cells.

### 2.2. Involvement of ADAM17 Activation in TGF-β Induced CTGF Expression in A549 Cells

**Figure 2:**
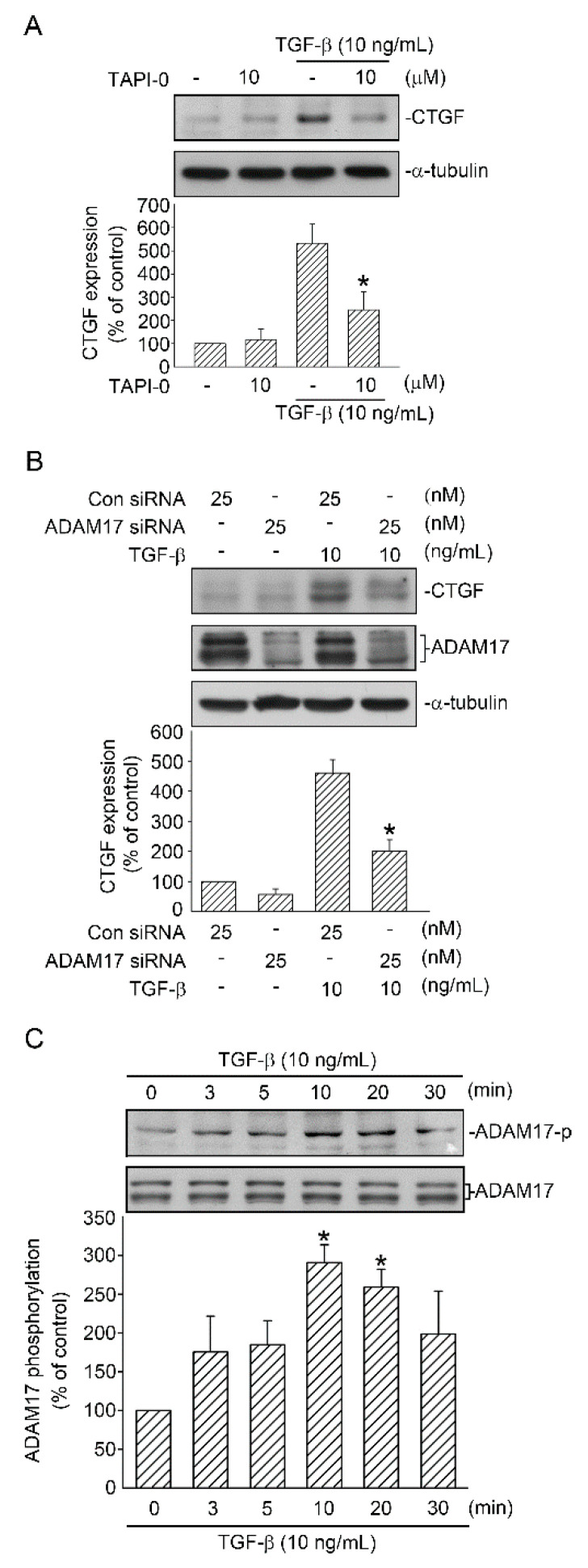
Participation of ADAM17 in TGF-β-induced CTGF expression in human lung epithelial A549 cells. (**A**) A549 cells were processed with the ADAM17 inhibitor TAPI-0 (10 μM) for 20 min. After 20 min, the cells were stimulated with TGF-β (10 ng/mL) for an additional 6 h. CTGF and α-tubulin levels were detected in cell lysates through Western blotting. Data are expressed as mean ± SEM of three independent experiments. * *p* < 0.05, compared with the TGF-β group without TAPI-0 treatment. (**B**) A549 cells were transfected with 25 nM control siRNA (con siRNA) and 25 nM ADAM17 siRNA for 48 h before they were stimulated with TGF-β (10 ng/mL) for an additional 6 h. CTGF and α-tubulin levels were detected in cell lysates through Western blotting. Results are expressed as mean ± SEM of three independent experiments. * *p* < 0.05, compared with TGF-β plus the control siRNA group. (**C**) Cells were stimulated with TGF-β (10 ng/mL) for 0–30 min, and then ADAM17 phosphorylation and ADAM17 were detected in cell lysates through Western blotting. Results are presented as mean ± SEM of four independent experiments. * *p* < 0.05, compared with the control group without TGF-β treatment.

ADAM17 participates in several pathways, including the TGF-β signaling pathway, and contributes to tissue fibrosis [7,8]. To determine whether ADAM17 mediates TGF-β-induced CTGF expression, an ADAM17 inhibitor (TAPI-0) was used. TAPI-0 can also inhibit matrix metalloprotease (MMP). Treatment of cells with TAPI-0 (10 µM) inhibited TGF-β-induced CTGF expression by 70.4% ± 18.3% (Figure 2A). In addition, when ADAM17 siRNA (25 nM) was used for a specific knockdown, ADAM17 siRNA attenuated TGF-β-induced CTGF expression by 60.1% ± 10.4% (Figure 2B). Furthermore, ADAM17 siRNA markedly inhibited ADAM17 protein expression (Figure 2B). We further investigated whether TGF-β induced ADAM17 activation. Phosphorylation of the ADAM17 Thr735 residue enhances cell surface presentation and the stability and activity of ADAM17 [21], and phosphorylated ADAM17 Thr735-specific antibody was used to investigate the phosphorylation of ADAM17. Stimulation of A549 cells with TGF-β (10 ng/mL) in a time-dependent manner induced an increase in ADAM17 Thr735 phosphorylation, and the peak response was achieved at 10 min (Figure 2C). These results indicated that ADAM17 was a downstream molecule of the TGF-β signaling pathway and regulated TGF-β-induced CTGF expression in A549 cells.

### 2.3. Mediating Effects of ERK on TGF-β-Induced CTGF Expression and ADAM17 Phosphorylation

**Figure 3:**
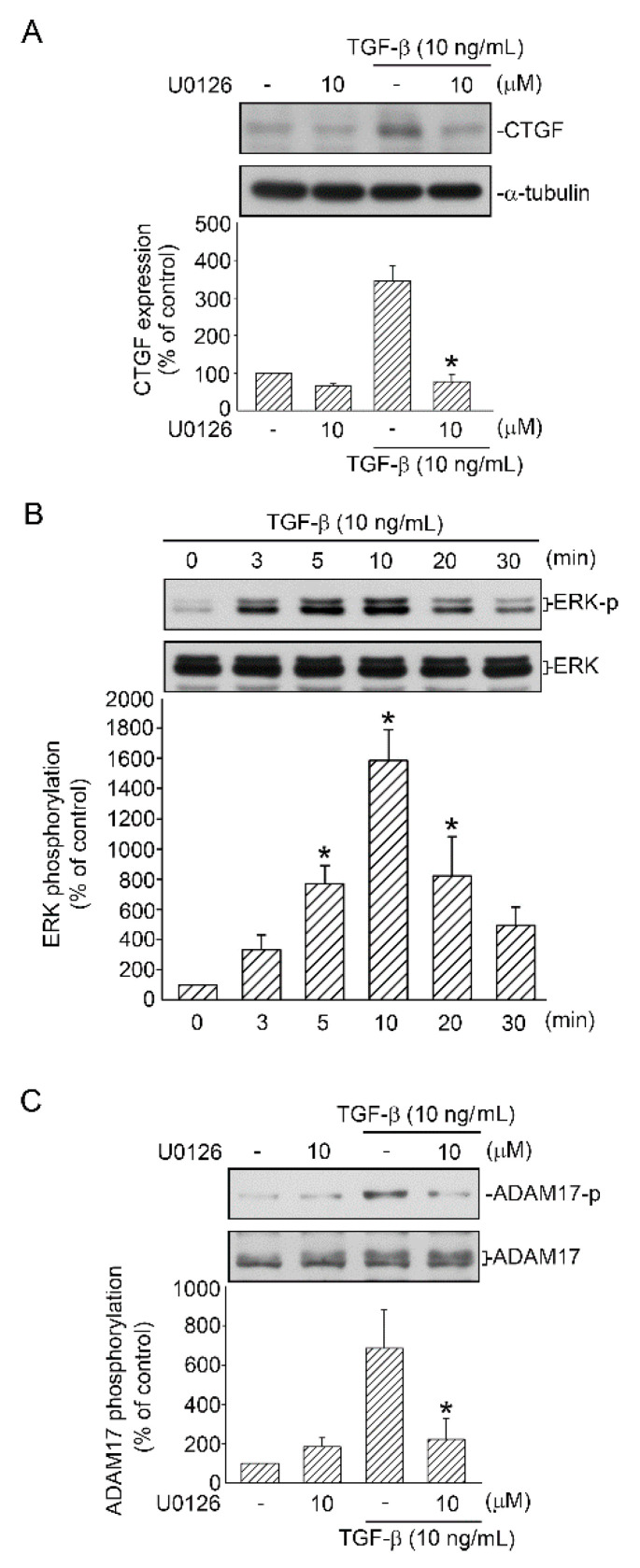
Participation of ERK in TGF-β-induced CTGF expression and ADAM17 activation in human lung epithelial A549 cells. (**A**) Cells were processed with the ERK inhibitor U0126 (10 μM) for 20 min before they were stimulated with TGF-β (10 ng/mL) for an additional 6 h. CTGF or α-tubulin in cell lysates were immunodetected with specific antibodies. Data are expressed as mean ± SEM of three independent experiments. * *p* < 0.05, compared with the TGF-β group without U0126 treatment. (**B**) A549 cells were stimulated with TGF-β (10 ng/mL) for 0–30 min, and then the levels of ERK phosphorylation and ERK in cell lysates were immunodetected with specific antibodies. Data are presented as mean ± SEM of four independent experiments. * *p* < 0.05, compared with the control group without TGF-β treatment. (**C**) Cells were treated with the ERK inhibitor U0126 (10 μM) for 20 min before they were stimulated with TGF-β (10 ng/mL) for an additional 10 min. Cell lysates were prepared, and specific antibodies for ADAM17 phosphorylation and ADAM17 were detected through Western blotting. Data are presented as mean ± SEM of three independent experiments. * *p* < 0.05, compared with the TGF-β group without U0126 treatment.

ERK is a key mediator of several TGF-β-regulated cellular functions [22]. To confirm whether ERK phosphorylation mediates TGF-β-induced CTGF expression, A549 cells were processed with 10 μM U0126 (an ERK inhibitor). Zhai et al. demonstrated that U0126 effectively inhibited the expression of p-ERK in A549 cells [23]. We further examine whether ERK participates in TGF-β-induced CTGF expression. After treatment with U0126 for 20 min, the cells were treated with TGF-β (10 ng/mL) for an additional 6 h. U0126 (10 μM) completely reduced TGF-β-induced CTGF expression (FIGURE 3A). Subsequently, an antibody specific for ERK phosphorylated at Tyr204 was used to define ERK phosphorylation. After A549 cells were processed with TGF-β (10 ng/mL) for 0–30 min, ERK Tyr204 phosphorylation increased at 5 min, achieved a peak at 10 min, and declined after 20 min of treatment (Figure 3B). A previous study suggested that ERK phosphorylates ADAM17 in kidney cells during profibrotic stimulation [24]. We evaluated the role of ERK in TGF-β-induced activation of ADAM17 in human lung epithelial cells. A549 cells were processed with U0126 (10 μM) for 20 min and then treated with TGF-β (10 ng/mL) for 10 min. As a result, ADAM17 phosphorylation was decreased (Figure 3C). This result implied that TGF-β activated ERK, which mediated TGF-β-induced CTGF expression and, in turn, might affect ADAM17 function.

### 2.4. RSK1-Mediated TGF-β-Induced CTGF Expression

**Figure 4:**
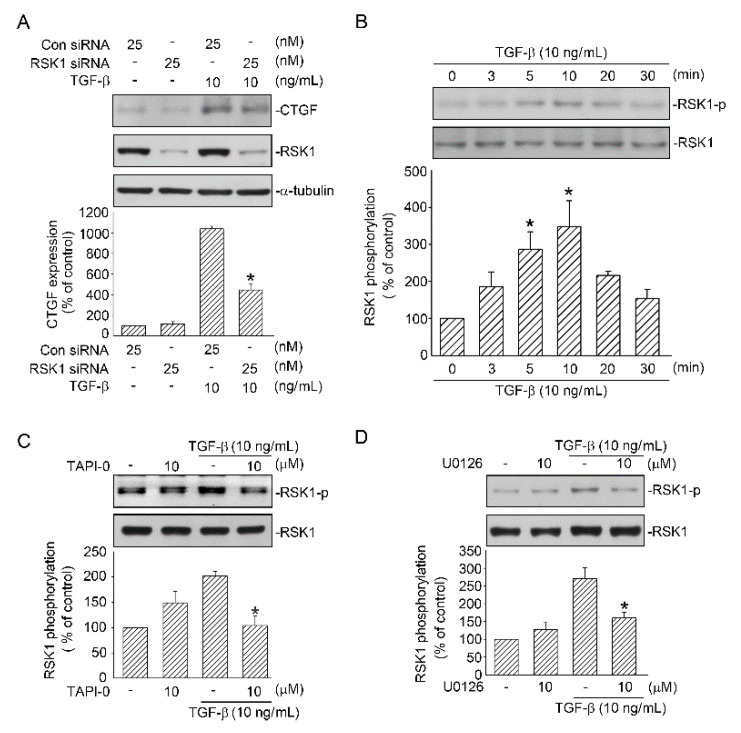
Involvement of RSK1 activation in TGF-β-induced CTGF expression through the ERK/ADAM17 pathway in human lung epithelial A549 cells. (**A**) Cells were transfected with 25 nM control siRNA (con siRNA) and 25 nM RSK1 siRNA for 48 h before they were stimulated with TGF-β (10 ng/mL) for an additional 6 h. Cell lysates were prepared, and specific antibodies for CTGF and α-tubulin were detected through Western blotting. Results are expressed as mean ± SEM of three independent experiments. * *p* < 0.05, compared with TGF-β plus the control siRNA group. (**B**) Cells were stimulated with TGF-β (10 ng/mL) for 0–30 min, and then the levels of RSK1 phosphorylation and RSK1 in cell lysates were immunodetected with specific antibodies. Results are presented as mean ± SEM of three independent experiments. * *p* < 0.05, compared with the control group without TGF-β treatment. (**C**) Cells were treated with the ADAM17 inhibitor TAPI-0 (10 μM) for 20 min before they were stimulated with TGF-β (10 ng/mL) for an additional 10 min. RSK1 phosphorylation and RSK1 in cell lysates were immunodetected with specific antibodies. Results are expressed as mean ± SEM of four independent experiments. * *p* < 0.05, compared with the TGF-β group without TAPI-0 treatment. (**D**) Cells were pretreated with U0126 (10 μM) for 20 min before they were stimulated with TGF-β (10 ng/mL) for an additional 10 min. RSK1 phosphorylation and RSK1 in cell lysates were immunodetected with specific antibodies. Results are presented as mean ± SEM of three independent experiments. * *p* < 0.05, compared with the TGF-β group without U0126 treatment.

Next, we confirmed whether RSK1 is involved in CTGF expression induced by TGF-β. TGF-β-induced CTGF expression was inhibited by 54.7% ± 9.6% after A549 cells were transfected with RSK1 siRNA (25 nM) (Figure 4A). Additionally, when the cells were transfected with RSK1 siRNA (25 nM), the RSK1 expression decreased, indicating that RSK1 siRNA was functional (Figure 4A). Next, to investigate TGF-β-induced phosphorylation of RSK1, we used an antibody specific for RSK1 phosphorylated at Ser221. Incubation of A549 cells with TGF-β (10 ng/mL) for 0–30 min time-dependently induced RSK1 phosphorylation, which increased at 5 min, reached a maximum at 10 min, and declined after treatment for 20 min (Figure 4B). Subsequently, we examined whether ADAM17 mediates TGF-β-induced RSK1 phosphorylation. Pretreatment of cells with TAPI-0 (10 μM) for 20 min attenuated TGF-β-induced RSK1 phosphorylation (Figure 4C). RSK1 is activated by the ERK signaling pathway [25], and, as mentioned above, ERK is involved in TGF-β-mediated CTGF expression in A549 cells. We then investigated whether RSK1 phosphorylation occurs through the ERK-mediated TGF-β signaling pathway and found that TGF-β-induced RSK1 phosphorylation was inhibited by U0126 (10 μM), and RSK1 phosphorylation decreased by 81.0% ± 9.2% (Figure 4D). These data revealed that in addition to ADAM17 and ERK, RSK1 is involved in TGF-β-induced CTGF expression.

### 2.5. Role of C/EBPβ in TGF-β-Induced CTGF 

**Figure 5:**
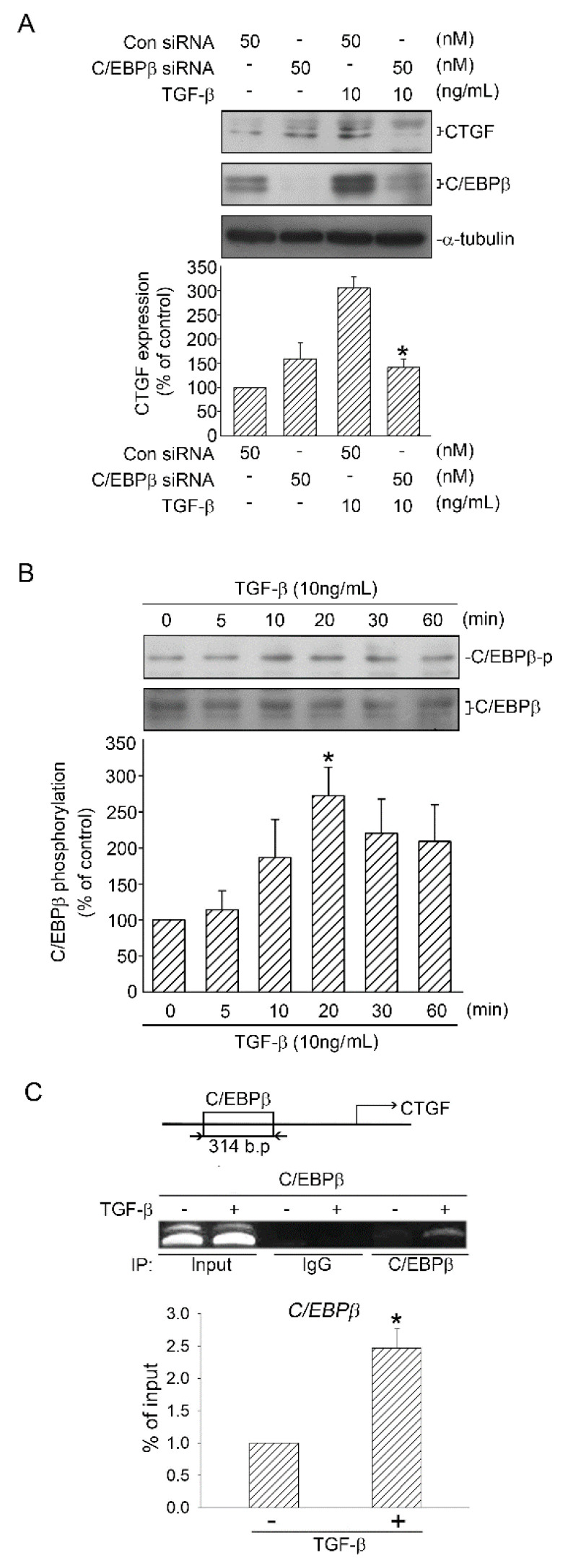
Participation of C/EBPβ in TGF-β-induced CTGF expression in human lung epithelial A549 cells. (**A**) A549 cells were transfected with 50 nM control siRNA (con siRNA) and C/EBPβ siRNA (50 nM) for 48 h before they were stimulated with TGF-β (10 ng/mL) for an additional 6 h. CTGF and α-tubulin levels were detected in cell lysates through Western blotting. Results are presented as mean ± SEM of four independent experiments. * *p* < 0.05, compared with TGF-β plus the control siRNA group. (**B**) Cells were stimulated with TGF-β (10 ng/mL) for 0–60 min, after which the levels of C/EBPβ phosphorylation and C/EBPβ in cell lysates were immunodetected with specific antibodies. Results are presented as mean ± SEM of five independent experiments. * *p* < 0.05, compared with the control group without TGF-β treatment. (**C**) Incubated cells were stimulated with TGF-β (10 ng/mL) for 20 min and detected using the ChIP assay, as mentioned in the “Materials and Methods” section. The typical traces indicated the three independent experiments that yielded similar results. Results are presented as mean ± SEM of three independent experiments. * *p* < 0.05, compared with the control group without TGF-β treatment.

Chiou et al. (2006) proved the existence of some transcription factor binding sites, including C/EBPβ, on the CTGF promoter [17]. To elucidate whether C/EBPβ participates in TGF-β-induced CTGF expression, C/EBPβ siRNA was used. When the cells were transfected with C/EBPβ siRNA (50 nM), TGF-β-induced CTGF expression was attenuated (Figure 5A). We then investigated C/EBPβ phosphorylation by using C/EBPβ Ser105 phosphorylated antibody. C/EBPβ phosphorylation increased and reached a peak at 20 min (Figure 5B). Next, we performed ChIP to ascertain the role of C/EBPβ in TGF-β-induced CTGF expression. We discovered that treatment of the cells with TGF-β (10 ng/mL) for 20 min enhanced the binding of C/EBPβ on the CTGF promoter, which exhibits a 2.5-fold increase in C/EBPβ-binding levels (Figure 5C). Taken together, these data suggested that C/EBPβ binds to the CTGF promoter when activated by TGF-β, which in turn induced CTGF expression.

### 2.6. Activation of C/EBPβ by TGF-β through ERK, ADAM17, and RSK1

**Figure 6:**
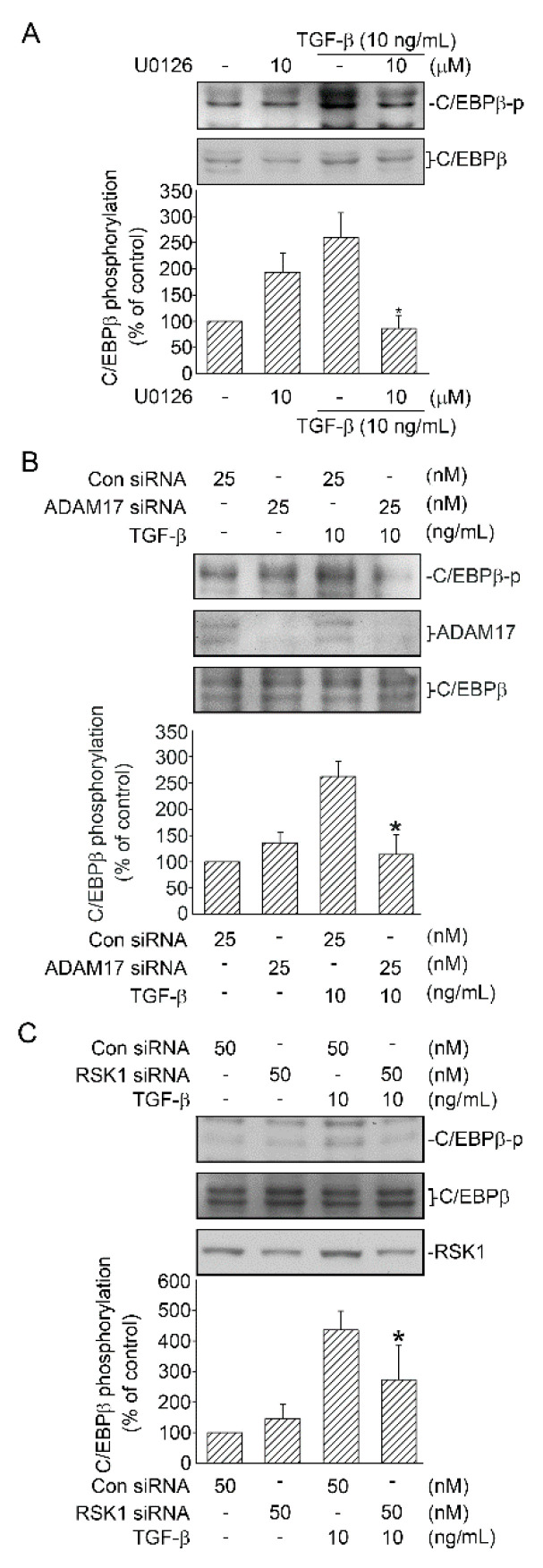
ERK, ADAM17, and RSK1 mediate TGF-β-induced C/EBPβ in human lung epithelial A549 cells. (**A**) A549 cells were processed with the ERK inhibitor U0126 (10 μM) for 20 min. After 20 min, the cells were treated with TGF-β (10 ng/mL) for an additional 20 min. C/EBPβ phosphorylation and C/EBPβ levels in cell lysates were immunodetected with specific antibodies. Data are expressed as mean ± SEM of three independent experiments. * *p* < 0.05, compared the TGF-β group without U0126 treatment. (**B**) A549 cells were transfected with control siRNA (con siRNA) and ADAM17 siRNA (25 nM). After 24 h, the cells were stimulated with TGF-β (10 ng/mL) for an additional 20 min. Cell lysates were prepared, and specific antibodies for C/EBPβ phosphorylation and C/EBPβ were immunodetected. Data are expressed as mean ± SEM of three independent experiments. * *p* < 0.05, compared with TGF-β plus the control siRNA group. (**C**) Cells were transfected with control siRNA (con siRNA) and RSK1 siRNA (50 nM) for 24 h before they were processed with TGF-β (10 ng/mL) for an additional 20 min. C/EBPβ phosphorylation and C/EBPβ were detected in cell lysates through Western blotting. Data are expressed as mean ± SEM of three independent experiments. * *p* < 0.05, compared with TGF-β plus the control siRNA group.

A recent study suggested that C/EBPβ can be phosphorylated by RSK1 in human lung epithelial cells [15]. As mentioned above, TGF-β-induced RSK1 phosphorylation was mediated by ERK and ADAM17. Thus, we treated A549 cells with U0126 (10 μM), TAPI-0 (10 μM), or RSK1 siRNA (50 nM) and found that phosphorylated C/EBPβ at Ser105 was attenuated, respectively (Figure 6A–C). These results implied that TGF-β-induced C/EBPβ was a downstream molecule of the ERK/ADAM17/RSK1 pathway.

### 2.7. TGF-β-Induced EMT Process in Human Lung Epithelial Cells

**Figure 7:**
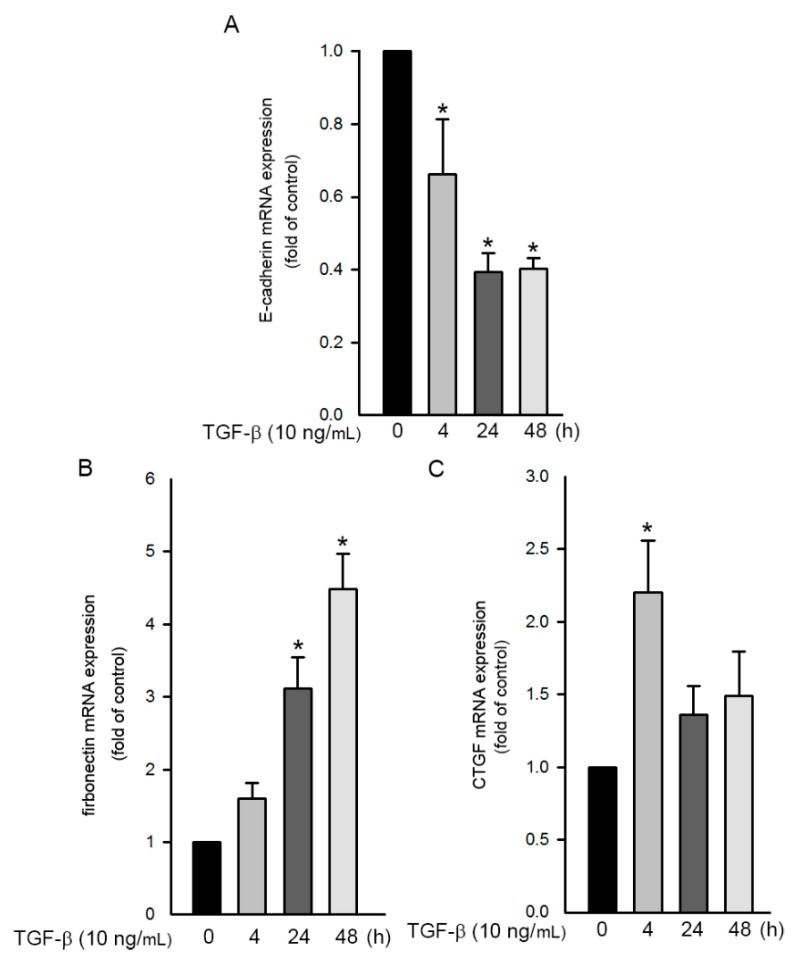
TGF-β-induced change in EMT-related mRNA levels in human lung epithelial A549 cells. (**A**) Cells were stimulated with TGF-β (10 ng/mL) for 0–48 h. Total RNA was isolated and E-cadherin mRNA was determined using qRT-PCR. Results are presented as mean ± SEM of three independent experiments. (**B**) Cells were stimulated with TGF-β (10 ng/mL) for 0–48 h. Total RNA was isolated, and FN mRNA was determined through qRT-PCR. Data are presented as mean ± SEM of three independent experiments. * *p* < 0.05, compared with the control group without TGF-β treatment. (**C**) Cells were stimulated with TGF-β (10 ng/mL) for 0–48 h. Total RNA was isolated, and *CTGF* mRNA was determined through qRT-PCR. Results are presented as mean ± SEM of seven independent experiments.

During EMT, TGF-β signaling pathway induces the expression of the growth-inhibited gene. The Smad-dependent transcriptional process then gradually attenuates the epithelial gene (such as E-cadherin) and activates the mesenchymal gene (such as FN) [3]. Therefore, we investigated the change in mRNA of the epithelial and mesenchymal genes through qPCR. After stimulation with TGF-β 10 ng/mL) for 0–48 h, the expression of E-cadherin and FN mRNA in A549 cells was examined. The results revealed that E-cadherin mRNA expression decreased (Figure 7A), whereas the expression of FN mRNA increased (Figure 7B). Moreover, we further found that TGF-β induced CTGF mRNA expression after 4h treatment (Figure 7C). According to these data, TGF-β induced CTGF expression as well as EMT in A549 cells.

### 2.8. Involvement of ADAM17 and CTGF in TGF-β-Induced FN Expression

**Figure 8:**
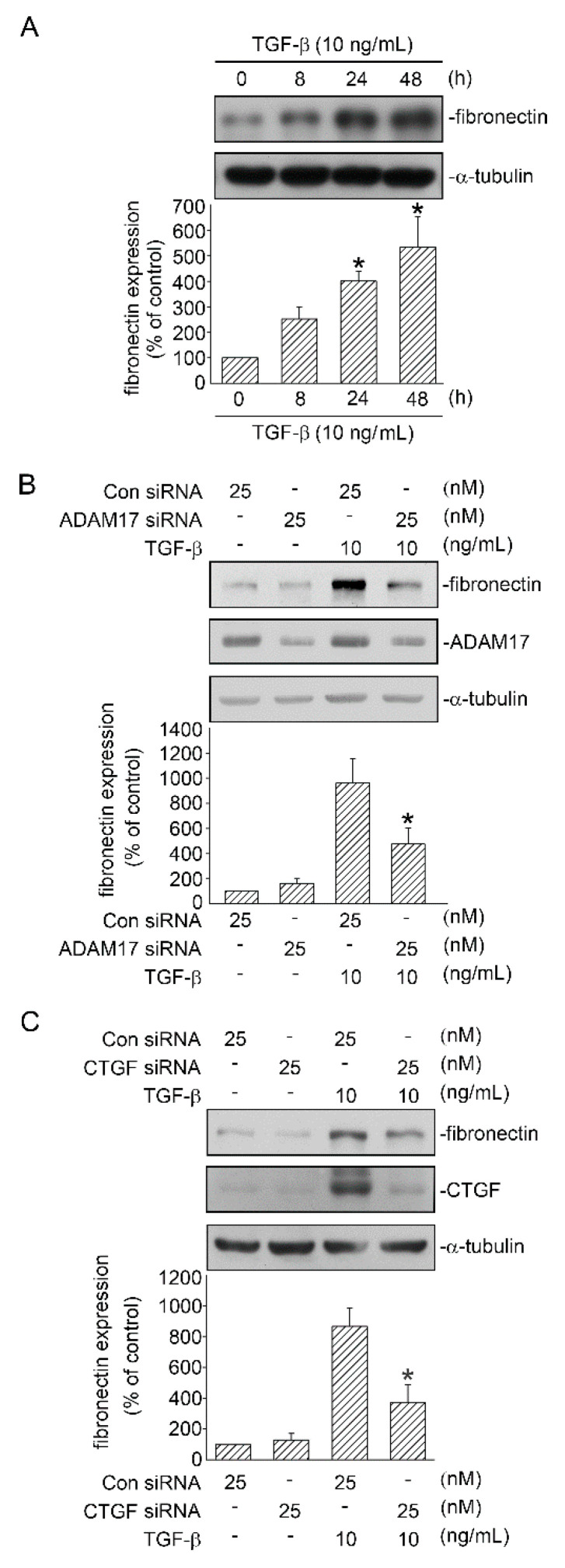
Involvement of ADAM17 and CTGF in TGF-β-induced FN expression in human lung epithelial A549 cells. (**A**) A549 cells were stimulated with TGF-β (10 ng/mL) for 0–48 h. FN and α-tubulin levels were detected in cell lysates through Western blotting. Data are presented as mean ± SEM of three independent experiments. * *p* < 0.05, compared with the control group without TGF-β treatment. (**B**) A549 cells were transfected with control siRNA (con siRNA) and ADAM17 siRNA (25 nM). After 24 h, the cells were stimulated with TGF-β (10 ng/mL) for an additional 48 h. FN and α-tubulin levels were detected through Western blotting. Results are presented as mean ± SEM of three independent experiments. * *p* < 0.05, compared with TGF-β plus the control siRNA group. (**C**) Cells were transfected with control siRNA (con siRNA) and CTGF siRNA (25 nM). After 24 h, the cells were stimulated with TGF-β (10 ng/mL) for an additional 48 h. FN and α-tubulin levels were detected through Western blotting. Results are presented as mean ± SEM of three independent experiments. * *p* < 0.05, compared with TGF-β plus the control siRNA group.

As mentioned above, FN is a mesenchymal marker that increased during TGF-β-induced EMT in human lung epithelial cells. In the present study, when A549 cells were stimulated with TGF-β 10 ng/mL) for 0–48 h, FN protein was expressed time-dependently and peaked at 48 h (Figure 8A). Next, we investigated whether ADAM17 is involved in TGF-β-induced FN protein expression. We discovered that treatment of cells with ADAM17 siRNA (25 nM) reduced TGF-β-induced FN protein expression (Figure 8B). In addition, we examined the role of CTGF in TGF-β-induced FN protein expression. Figure 8C shows that CTGF siRNA (25 nM) inhibited FN expression. These data indicated that ADAM17 and CTGF regulated TGF-β-induced expression of FN in human lung epithelial cells.

## 3. Discussion

ADAM17 is a crucial converting enzyme involved in numerous cellular functions such as EMT. Increased ADAM17 expression is identified in several inflammatory diseases, cancers, and organ fibrotic changes including pulmonary fibrosis [6,7,24,26,27]. During the progression of chronic pulmonary fibrosis, the volume and ventilation of the lungs are gradually decreased due to abnormal proliferation of fibroblasts through the EMT process, which causes collagen deposition and finally leads to architectural distortion [28,29,30]. Blom et al. (2002) suggested that CTGF appeared to play the role of a modulator and comediator of biological functions induced by TGF-β, including fibrogenesis [9]. In addition, several studies have shown that EMT-like changes are induced by the CTGF expression in lung epithelial cells. Furthermore, both EMT and differentiation of fibroblasts to myofibroblasts could be stimulated by TGF-β [9,14]. Therefore, CTGF and EMT play major roles in TGF-β-induced lung fibrosis. Recently, Chang et al. (2015) proved that in stem cells from the apical papilla, MEK/ERK and ALK5/smad2 signaling pathways were activated by TGF-β, which influenced the collagen content, cell growth, and ALP activity [22]. Kim et al. (2007) confirmed that RSK1 was a downstream mediator of ERK and phosphorylated the rat form of C/EBPβ at Ser105 in the N-terminal domain [16]. Moreover, a study demonstrated that the CTGF promoter might be activated by C/EBPβ after treatment with insulin-like growth factor-I in the zebrafish liver cell line [17]. In the present study, we found that ERK, ADAM17, RSK1, and C/EBPβ participated in acute TGF-β-induced CTGF expression in human lung epithelial cells. Additionally, ADAM17 and CTGF mediated TGF-β-induced FN expression. The results suggested that these acute TGF-β-mediated factors might play a crucial role in the expression of CTGF and EMT. Identifying the pathway involved in ADAM17-mediated TGF-β-induced expression of CTGF and EMT in human lung epithelial cells is crucial.

Numerous studies have confirmed that ADAMs, especially ADAM17, play a major role in modulating tumor growth and metastasis through regulating cell signaling pathways. In the progression of carcinomas, epithelial cells lose their characteristics, which are substituted by those of mesenchymal cells through the EMT process that is induced by ECM and TGF-β. In addition, Malapeira et al. (2011) proved that ADAM17 regulated the TGF-β signaling pathway through the cleavage of vasorin and finally modulated EMT [7,27]. ADAM17 participates in pulmonary fibrosis by inducing ACE-2 ectodomain shedding [8]. However, the relationship between ADAM17 and TGF-β-induced CTGF expression remains unclear. In the present study, treating cells with ADAM17 siRNA attenuated the TGF-β-induced CTGF expression in A549 cells. In addition, treatment with ADAM17 siRNA caused a decrease in the FN expression in A549 cells stimulated with TGF-β. Studies have shown that increased CTGF expression is investigated in patients with bronchopulmonary dysplasia and pulmonary fibrosis. Furthermore, CTGF expression might induce EMT-like changes in the adjacent epithelial cells when CTGF is overexpressed in lung fibroblasts [14,31,32]. The results of the present study showed that transfection of cells with CTGF siRNA reduced TGF-β-induced FN expression. Taken together, the results implied that ADAM17 and CTGF played crucial roles in TGF-β-induced FN expression in human lung epithelial cells. 

The ERK pathway plays a major role in the progression of pulmonary fibrosis through regulating osteopontin, which is recognized as a proinflammatory and profibrotic cytokine and is usually activated by growth factors through their receptor tyrosine kinases. Moreover, ERK1/2 is highly activated in the lung tissue of a patient with early stage IPF [33,34]. Yang et al. (2016) suggested that in mice with schistosomiasis hepatic fibrosis, CTGF was induced by corilagin through the miR-21/Smad7/ERK signaling pathway [35]. Because RSK1 is a member of MAPKAPK, which are modulated by ERK and p38 MAPKs [36], we questioned whether RSK1 plays a role in ERK-mediated TGF-β-induced CTGF expression. In the present study, we found that treatment with the ERK inhibitor U0126 attenuated TGF-β-induced CTGF expression in A549 cells, and so did treatment with RSK1 siRNA. Additionally, stimulation with TGF-β enhanced the phosphorylation of ERK and RSK1. Moreover, TGF-β-induced RSK1 phosphorylation decreased in the cells treated with U0126. Thus, ERK activation was involved in RSK1 phosphorylation and TGF-β-induced CTGF expression. A previous study showed that the expressed pro-ADAM17 is phosphorylated by ERK in intact cells [37]. To further examine the role of ADAM17 in the ERK/RSK1 pathway, we used the ERK inhibitor U0126 and ADAM17 inhibitor TAPI-0 to treat A549 cells and found that ADAM17 phosphorylation and RSK1 phosphorylation were inhibited, respectively, suggesting that ERK mediated TGF-β-induced ADAM17 phosphorylation, and ADAM17 regulated TGF-β-induced RSK1 phosphorylation. Overall, our results demonstrated that TGF-β enhanced CTGF expression through the ERK/ADAM17/RSK1 signaling pathway.

**Figure 9:**
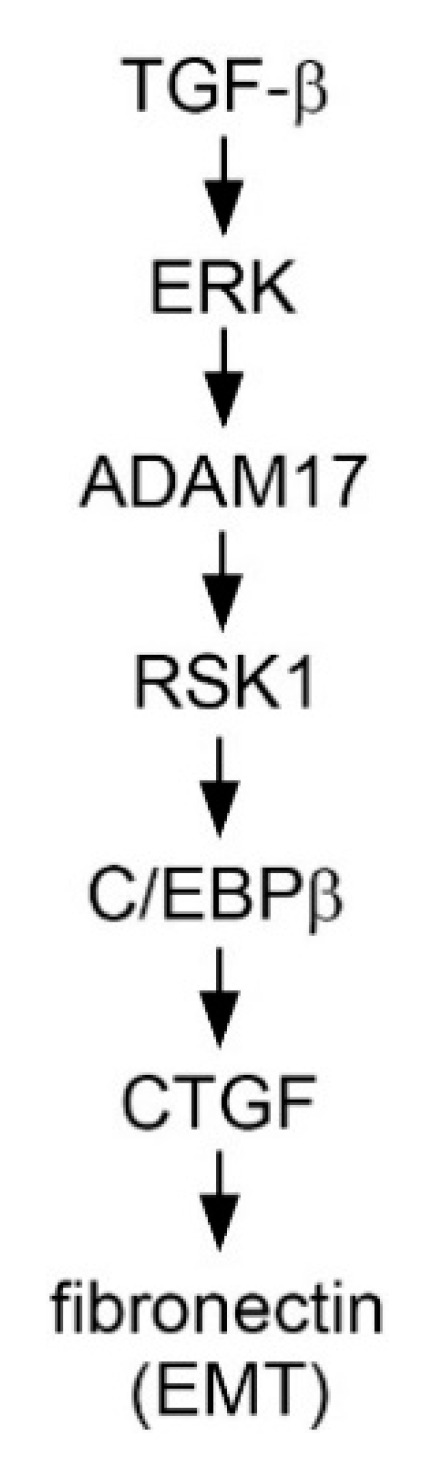
Simplified image displaying the results of TGF-β-induced CTGF expression mediated via the ERK/ADAM17/RSK1/C/EBPβ pathway in human lung epithelial cells. TGF-β activated the ERK/ADAM17/RSK1/C/EBPβ signaling pathway, which in turn initiates binding of C/EBPβ to the CTGF promoter and ultimately induces CTGF expression in human lung epithelial cells. Moreover, ADAM17 and CTGF participated in TGF-β-induced FN expression.

Chiou et al. (2006) suggested that the CTGF proximal promoter sequence included TATAA box, putative AP-1, C/EBPα, and C/EBPβ binding sites [17]. In addition, the promoter of the human collagen I gene has a binding site for C/EBPβ. Collagen I is recognized as a crucial mediator for TGF-β- and CTGF-induced fibrogenic processes [38]. However, the role of C/EBPβ in TGF-β-induced CTGF expression remains unclear. In the present study, we found that C/EBPβ markedly bound to the CTGF promoter upon TGF-β stimulation. Furthermore, when the cells were transfected with C/EBPβ siRNA, TGF-β-induced CTGF expression was inhibited, demonstrating that C/EBPβ exerted a positive modulation on TGF-β-induced CTGF expression. We also found that treatment with U0126, ADAM17 siRNA, and RSK1 siRNA inhibited C/EBPβ phosphorylation, suggesting that TGF-β induced C/EBPβ activation through the ERK/ADAM17/RSK1 pathway. Furthermore, the results of our study indicated that ADAM17 played a master role in TGF-β-induced CTGF expression and EMT through the ERK/RSK1/C/EBPβ pathway. In conclusion, TGF-β activated the ERK/ADAM17/RSK1/C/EBPβ signaling pathway, after which it promoted the connection of C/EBPβ to the C/EBPβ site on the CTGF promoter region to regulate CTGF expression in human lung epithelial cells. Moreover, ADAM17 and CTGF participated in TGF-β-induced FN expression. Our results reveal a signaling pathway related to ADAM17, CTGF, and FN and can be used to interpret how TGF-β induces the expression of CTGF and EMT, which may provide a new orientation for the treatment of IPF (Figure 9).

## 4. Materials and Methods

### 4.1. Materials

The human lung epithelial cell line (A549) (Cat# CCL-185, RRID:CVCL_0023) was purchased from the American Type Culture Collection (Manassas, VA, USA). Dulbecco’s Modified Eagle’s medium/Ham’s F-12, fetal calf serum, and penicillin/streptomycin were obtained from Invitrogen Life Technologies (Carlsbad, CA, USA). TGF-β was purchased from PeproTech (London, UK). U0126 was acquired from Calbiochem-Novabiochem (San Diego, CA, USA). TNF-α processing inhibitor-0 (TAPI-0) was obtained from Enzo Life Science (Farmindale, NY, USA). Lipofectamine 3000 and Lipofectamine Plus reagent as well as a minimum essential medium were purchased from Invitrogen Life Technologies. C/EBPβ small interfering RNA (siRNA), RSK1 siRNA, ADAM17 siRNA, and scrambled siRNA (control) were acquired from Sigma-Aldrich (St. Louis, MO, USA). An α-tubulin antibody (Cat# T5168, RRID:AB_477579) was obtained from Sigma-Aldrich (St. Louis, MO, USA). Antibodies specific for RSK1 (Cat# sc-231, RRID:AB_632367) ERK (Cat# sc-94, RRID:AB_2140110), ERK phosphorylated at Tyr204 (Cat# sc-7383, RRID:AB_627545), CTGF (Cat# sc-14939, RRID:AB_638805), and C/EBPβ Cat# sc-7962, RRID:AB_626772; and anti-mouse (Cat# sc-2005, RRID:AB_631736), anti-goat (Cat# sc-2020, RRID:AB_631728), and anti-rabbit (Cat# sc-2004, RRID:AB_631746) IgG-conjugated horseradish peroxidase antibodies, were obtained from Santa Cruz Biotechnology (Santa Cruz, CA, USA). Antibodies specific for ADAM17 (Cat# ab2051, RRID:AB_302796), ADAM17 phosphorylation at Thr735 (Cat# ab195828, RRID:AB_2750979), RSK1 phosphorylation at Ser221 (Cat# sc-231, RRID:AB_632367), and fibronectin (Cat# ab2413, RRID:AB_2262874) were purchased from Abcam (Cambridge, MA, USA). An antibody specific for C/EBPβ phosphorylated at Ser105 antibody (Cat# Ap20701b, RRID:AB_2750980) was acquired from Abgent (San Diego, CA, USA). A nucleospin RNA kit was obtained from Macherey-Nagel (Bethlehem, PA, USA). A chromatin immunoprecipitation (ChIP) assay kit was acquired from Upstate Biotech Millipore (Lake Placid, NY, USA). All materials required for Western blotting were obtained from Bio-Rad (Hercules, CA, USA). 

### 4.2. Cell Culture

Human lung epithelial (A549) cells were cultured as described previously [15]. In brief, A549 cells were seeded in Dulbecco’s Modified Eagle’s medium/Ham’s F-12 nutrient mixture, containing 10% fetal calf serum, 100 U/mL penicillin, 250 μg/mL fungizone, and 100 μg/mL streptomycin, in a humidified incubator at 37 °C. Cells were passaged onto 60-mm dishes for Western blotting and qRT-PCR or onto 100-mm dishes for the ChIP assay after achieving confluence.

### 4.3. siRNAs Transfection

A549 cells (2 10^5^ cell/well) were transfected with scrambled siRNA, ADAM17 siRNA, RSK1 siRNA, C/EBPβ siRNA, and CTGF siRNA with lipofectamine 3000 reagents for 24 h according to the manufacturer’s instructions. The medium was suctioned and substituted with fresh growth medium after siRNA transfection and then treated with TGF-β for the indicated time intervals. 

### 4.4. Western Blotting 

A549 cells were treated with TAPI-0 or U0126 for 20 min or transfected with control siRNA, ADAM17 siRNA, RSK1 siRNA, C/EBPβ siRNA, or CTGF siRNA. After 24 h, cells were treated with TGF-β. Immunoreactivity was assessed through Western blotting as described previously [15].

### 4.5. Chromatin Immunoprecipitation Assay

A549 cells were stimulated with TGF-β (10 ng/mL). After 20 min, they were fixed with formaldehyde for an additional 10 min. The C/EBPβ binding to the promoter region of CTGF was investigated using the ChIP assay, performed as previously described [15]. The primer sequences for PCR amplification on the promoter site of CTGF were C/EBPβ, sense: 5′-AAT CAG GAG TGG TGC GAA GA-3′ and antisense: 5′-AGC GGG GAA GAG TTG TTG TG-3′.

### 4.6. RNA Isolation and RT-PCR

According to the instructions, RNA was isolated from A549 cells. By using Moloney Murine Leukemia Virus Reverse Transcriptase (Toronto, ON, Canada), total RNA (1 μg) was isolated for RT. Subsequently, 10 μg cDNA of FN, CTGF, and E-cadherin was amplified using SYBR Green with a Rotor Gene Q system to investigate changes in mRNA. Primer sequences for FN were forward, 5′-ACC CAA TTC CTT GCT GGT ATC A-3′, and reverse, 5′-GTA TAT TCG GTT CCC GGT TCC-3′. The CTGF primer sequences were forward, 5′-CTG GCG GCT TAC CGA CTG-3′, and reverse, 5′-GGC TCT GCT TCT CTA GCC TG-3′. Primer sequences of E-cadherin were forward, 5′-GAG AAA CAG GAT GGC TGA AGG-3′, and reverse, 5′-TGA GGA TGG TGT AAG CGA TGG-3′. Primer sequences of β-actin for the internal control were forward, 5′-CCA ATC TGC TGG AAG GTG G-3′, and reverse, 5′-GAC TAC CTC ATG AAG ATC CT-3′.

### 4.7. Statistical Analysis

Data are presented as the mean ± standard error of the mean (SEM) of at least three independent experiments. One-way analysis of variance followed by Dunnett’s test was conducted to define diversities between means. A *p* value of <0.05 was considered statistically significant.

## Abbreviations

ADAM17	A disintegrin and metalloproteinase 17
ANOVA	Analysis of variance
C/EBPβ	CCAAT/enhancer-binding protein β
ChIP	Chromatin immunoprecipitation
CTGF	Connective tissue growth factor
ECM	Extracellular matrix
EMT	Epithelial–mesenchymal transition
ERK	Extracellular signal-regulated kinase
FN	Fibronectin
IPF	Idiopathic pulmonary fibrosis
RSK	Ribosomal S6 kinases 1
siRNA	Small interfering RNA
TGF-β	Transforming growth factor-β
